# Early Moderate Intensity Aerobic Exercise Intervention Prevents Doxorubicin-caused Cardiac Dysfunction through Inhibition of Cardiac Fibrosis and Inflammation

**DOI:** 10.3390/cancers12051102

**Published:** 2020-04-28

**Authors:** Hsin-Lun Yang, Pei-Ling Hsieh, Ching-Hsia Hung, Hui-Ching Cheng, Wan-Ching Chou, Pei-Ming Chu, Yun-Ching Chang, Kun-Ling Tsai

**Affiliations:** 1 Department of Physical Therapy, College of Medicine, National Cheng Kung University, Tainan 701, Taiwan; t66064043@pt.ncku.edu.tw (H.-L.Y.); chhung@mail.ncku.edu.tw (C.-H.H.); qaz200408@gmail.com (H.-C.C.); jinjaychou@gmail.com (W.-C.C.); 2 Department of Anatomy, School of Medicine, China Medical University, Taichung 404, Taiwan; plhsieh@mail.cmu.edu.tw (P.-L.H.); pmchu@mail.cmu.edu.tw (P.-M.C.); 3 Institute of Allied Health Sciences, College of Medicine, National Cheng Kung University, Tainan 701, Taiwan; 4 Department of Nursing, Shu-Zen Junior College of Medicine and Management, Kaohsiung 821, Taiwan; ychang@ms.szmc.edu.tw

**Keywords:** doxorubicin, cardiac toxicity, cardiomyocytes, cardiac fibrosis

## Abstract

Doxorubicin (DOX) is known as an effective drug in the fight against various cancers. However, one of the greatest impediments is DOX-induced cardiomyopathy, which may potentially lead to heart failure. Accumulating evidence has shed light on the pathological mechanism of DOX-induced cardiotoxicity, but treatments to mitigate the cardiac damage are still required. In an attempt to address this issue, we evaluated whether exercise provides cardioprotective effects on the DOX-induced cardiotoxicity. We showed that treadmill exercise (3 times/week; 1-week of exercise acclimatization and 4-weeks of endurance exercise) during the DOX treatment successfully prevented the cardiac dysfunction. The DOX-stimulated expression of IκBα, NF-κB, COX-2, and IL-8 were all downregulated by exercise as well as the fibrosis factors (TGF-β1, phosphorylated ERK, Sp1, and CTGF). Moreover, we showed that treadmill exercise diminished the expression of several cardiac remodeling-associated factors, such as FGF2, uPA, MMP2, and MMP9. These results were in line with the finding that exercise intervention reduced cardiac fibrosis and restored cardiac function, with higher values of ejection fraction and fractional shortening compared to the DOX-treated group. Two commonly used indicators of cardiac injury, lactate dehydrogenase, and creatine kinase-MB, were also decreased in the exercise group. Collectively, our results suggested that it may be beneficial to prescribe treadmill exercise as an adjunct therapy to limit cardiac damage caused by DOX.

## 1. Introduction

Doxorubicin (DOX), also known by the trade names Adriamycin^®^ or Rubex^®^, is an anthracycline antibiotic that serves as an efficacious chemotherapeutic agent in a broad spectrum of cancers. Although the mechanisms underlying the action of DOX in cancer cells remain controversial, it is considered that DOX causes the generation of free radicals, leading to DNA damage or lipid peroxidation. The initiation of DNA damage via the inhibition of topoisomerase II results in the induction of apoptosis [1]. Despite the broad clinical indications, chronic administration of DOX causes adverse effects. DOX-induced cardiomyopathy, such as congestive heart failure (CHF), can ultimately be a lethal disease [2,3]. A report by Swain et al. suggested that CHF may occur at a total cumulative DOX dose of 300 mg/m^2^, and dependent upon the cumulative dose, an estimated percentage of DOX-related CHF was around 26% of patients at 550 mg/m^2^, and 48% of patients at 700 mg/m^2^ [3]. Hence, approaches to alleviate cardiotoxicity and prevent DOX-induced CHF are of great interest. 

As with other forms of dilated cardiomyopathy, hearts affected by DOX cardiotoxicity exhibit similar morphologic and echocardiographic features. Histologically, DOX-induced cardiomyopathy is characterized by extensive fibrosis [2]. Functionally, the contractile capacity is reduced with a concomitant diastolic dysfunction [3]. Multifactorial pathways are implicated in the molecular mechanisms underlying the DOX-induced cardiotoxicity, such as nuclear factor kappa B (NF-κB) [4] and extracellular signal-regulated kinase (ERK1/2) [5]. It is known that the cumulative oxidation of cardiac mitochondrial DNA may contribute to irreversible cardiomyopathy [6]. Also, the accumulation of oxidative stress upregulated NF-κB [4] and consequently elevated the release of proinflammatory cytokines [7] and enhanced apoptosis [4] in the myocardium. A recent study has revealed that endurance exercise provides cardioprotection against DOX-induced cardiotoxicity through the reduction of apoptosis and upregulation of basal auto/mitophagy [8]. Their results showed that exercise prevented DOX-induced oxidative damages by suppressing NADPH oxidase 2 subunits [8]. This evidence, regarding the cardioprotective effect of exercise on DOX-related cardiotoxicity, prompted us to examine whether an exercise intervention could improve the morphologic and echocardiographic changes and other critical molecules that were associated with DOX-elicited cardiomyopathy. 

The positive clinical effects of an exercise intervention on cardiac function have been well addressed [9]. Jensen et al. found 10 weeks of exercise training before DOX administration helped to prevent DOX-induced damage [10]. Fourteen weeks of preconditioning exercise training reduces DOX-caused oxidative stress and oxidative injuries [11]. A previous study also suggested that acute preconditioning treadmill training protects against DOX-impaired function [12]. However, the cardiac protective effects of real-time exercise training on DOX-treated animals are still unclear. In the current study, we assessed the expression of NF-κB and several proinflammatory cytokines for the DOX injection combined with the exercise group. Subsequently, the expression levels of fibrosis and cardiac remodeling-related molecules were examined. Additionally, we investigated whether the morphologic and echocardiographic changes were improved by exercise treatment, as well as the concentration of cardiac enzymes that were used to indicate a cardiac injury. Results from this study will provide new evidence that exercise still has positive effects on cancer treatment if they have not been preconditioned to exercise training. 

## 2. Results

### 2.1. Early Moderate-Intensity Aerobic Exercise Ameliorates the DOX-Induced AKT Inhibition and Activation of Inflammatory Response 

**Figure 1:**
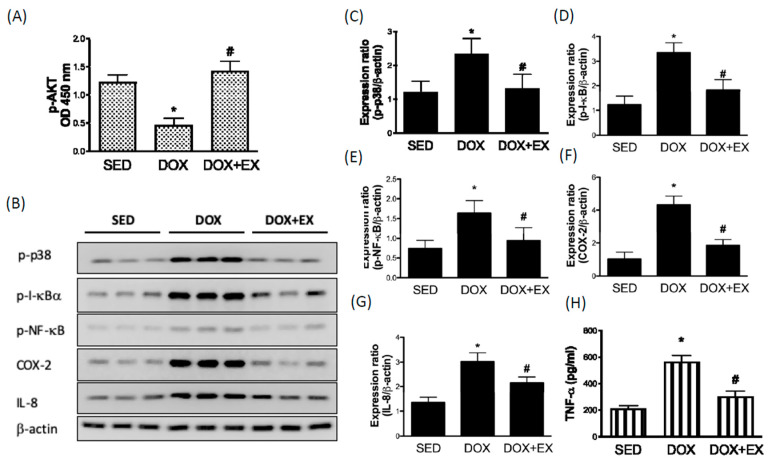
The effect of treadmill exercise on the DOX-induced AKT inhibition and up-regulation of inflammatory responses The phosphorylated AKT levels in cardiac tissues were investigated by ELISA (**A**). Representatives of Western blot (**B**), and the densitometric analysis of phosphorylated p38 (**C**), IκBα (**D**), NF-κB (**E**), COX-2 (**F**), and IL-8 (**G**). TNF-α levels were investigated by ELISA (**H**). * *p* < 0.05 compared to the SED group. # *p* < 0.05 compared to the DOX group. Detailed information about Figure 1B can be found at Figure S1.

Previous studies suggest that activation of AKT restores myocardial injuries in response to DOX treatment, whereas AKT repression promotes DOX-caused cardiac dysfunction [13,14]. Moreover, AKT activation is thought of as an effective strategy in the prevention of DOX-induced cardiac injuries [15]. We confirmed that DOX injection inhibited phosphorylated AKT expression in cardiac tissues, however, treadmill intervention restored this finding (Figure 1A). Activation of the p38 MAPK/NF-κB pathway contributes to DOX-caused cardiac inflammation through AKT inhibition has been addressed [7]. In addition, it is known that NF-κB is sequestered in the cytoplasm through direct interaction with its inhibitor proteins such as I-κBα. Subsequent to the activation of the I-κB kinase (IKK) complex, IκBα will be phosphorylated by IKK complex and degraded, allowing NF-κB to translocate to the nucleus and induce transcription of its target genes, such as proinflammatory cytokines [16]. As shown in Figure 1, we observed that DOX upregulated the phosphorylation of p38, IκBα, and NF-κB (Figure 1B–E), whereas treadmill intervention inhibited this phenomenon. The increased expression of cyclooxygenase-2 (COX-2) (Figure 1F) was in line with a previous study, which showed that DOX induced COX activity in rat neonatal cardiomyocytes [17]. Interleukin (IL)-8 and TNF-α are critical molecules that arre associated with cardiovascular diseases [18], and we showed that DOX increased the expression of IL-8 and TNF-α (Figure 1G,H). With the exercise treatment, the expression levels of COX-2 and IL-8 were both downregulated (Figure 1F,H).

### 2.2. Early Moderate-Intensity Aerobic Exercise Attenuates the DOX-Activated Fibrosis Factors

**Figure 2:**
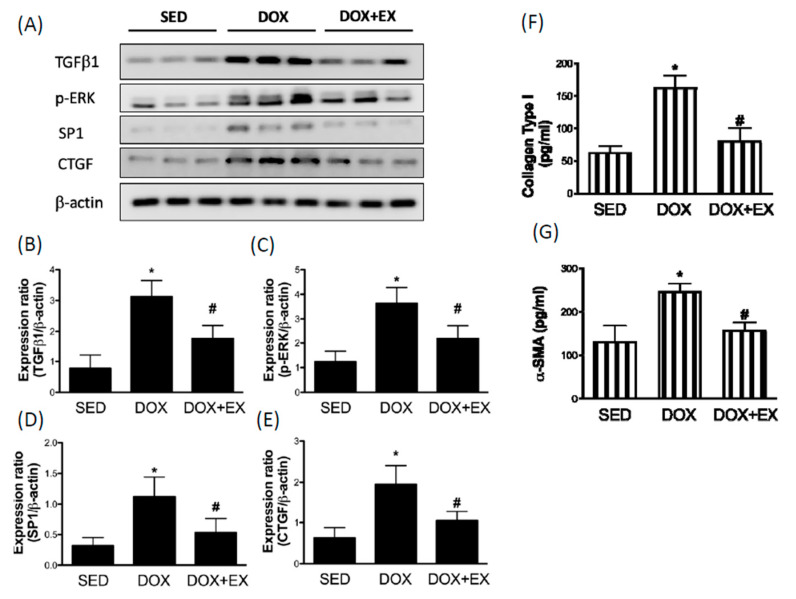
The effect of treadmill exercise on the DOX-driven upregulation of fibrosis factors Representatives of Western blot (**A**) and the densitometric analysis of TGF-β1 (**B**), phosphorylated ERK (**C**), Sp1 (**D**), and CTGF (**E**). The collagen type I (**F**) and α -SMA (**G**) were tested by ELISA. * *p* < 0.05 compared to the SED group. # *p* < 0.05 compared to the DOX group. Detailed information about Figure 2A can be found at Figure S2.

DOX directly induces the fibrotic response in the heart at the initial stage by up-regulation of proinflammatory events [19]. TGF-β is a key cytokine that plays an integral role in the development of fibrosis. It has long been known that TGF-β1 is able to activate ERK in fibroblasts [20], and that activation of ERK is required for TGF-β1-associated epithelial-to-mesenchymal transition [21], an important process for pathologic fibrosis. Our results showed that DOX upregulated the expression of TGF-β1 and phosphorylated ERK (Figure 2A–C), while exercise prevented this upregulation. In addition, we observed the induction of Sp1 (Figure 2D) and connective tissue growth factor (CTGF) (Figure 2E) by DOX treatment. Sp1 participated in TGF-β1-stimulated alpha 2(I)-collagen transcription [22], and CTGF was involved in the regulation of cardiac fibrosis and heart failure [23]. The increased expression levels of these two factors were also attenuated by exercise intervention (Figure 2D,E). These results were in line with the finding that exercise reduced canonical pro-fibrotic genes such as collagen type I and α -smooth muscle actin (α-SMA) levels in DOX-treated animals (Figure 2F,G).

### 2.3. Early Moderate-Intensity Aerobic Exercise Diminishes the DOX-Increased the Myocardial Remodeling-Associated Molecules 

**Figure 3:**
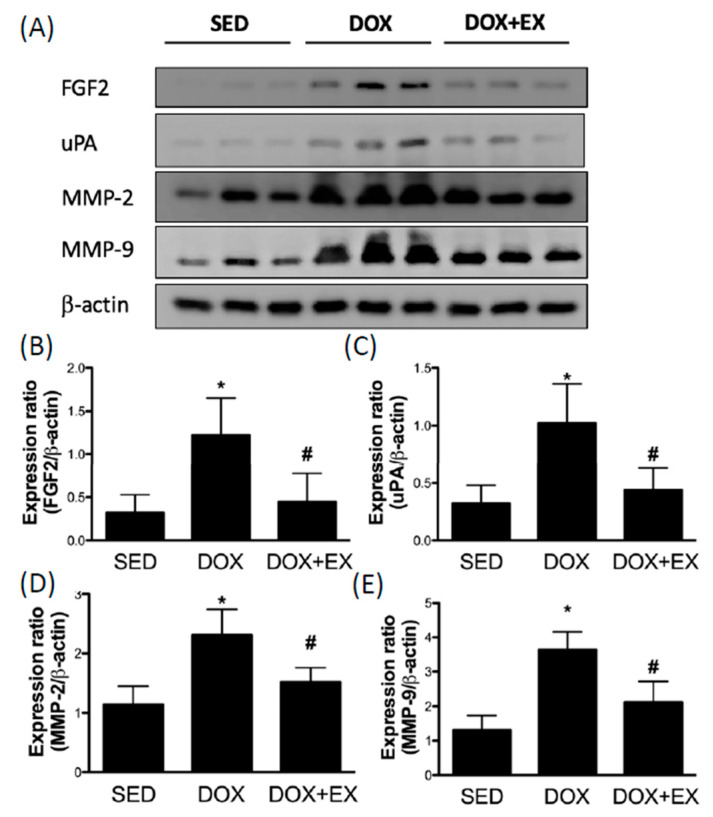
The effect of treadmill exercise on the DOX-triggered upregulation of cardiac remodeling-related factors Representatives of Western blot (**A**) and the densitometric analysis of FGF2 (**B**), uPA (**C**), MMP-2 (**D**) and MMP-9 (**E**). * *p* < 0.05 compared to the SED group. # *p* < 0.05 compared to the DOX group. Detailed information about Figure 3A can be found at Figure S3.

To further investigate the effect of DOX on cardiac remodeling, we examined two molecules that are associated with this process. Accumulation of fibroblast growth factor 2 (FGF-2) has been shown to exacerbate cardiac hypertrophy [24], and we demonstrated that DOX treatment increased the expression of FGF-2 (Figure 3A,B). Besides, the proteolytic enzymes urokinase-type plasminogen activator (uPA) and matrix metalloproteinases (MMPs) were both implicated in cardiac repair and remodeling [25]. As expected, DOX injection upregulated the expression levels of uPA, MMP2, and MMP9 (Figure 3C–E). Treadmill exercise lessened the induction of FGF-2, uPA, MMP2, and MMP9 by DOX administration and was consistent with the abovementioned results (Figure 3A–E).

### 2.4. Early Moderate-Intensity Aerobic Exercise Protects the Heart from the Damage Caused by DOX 

**Figure 4:**
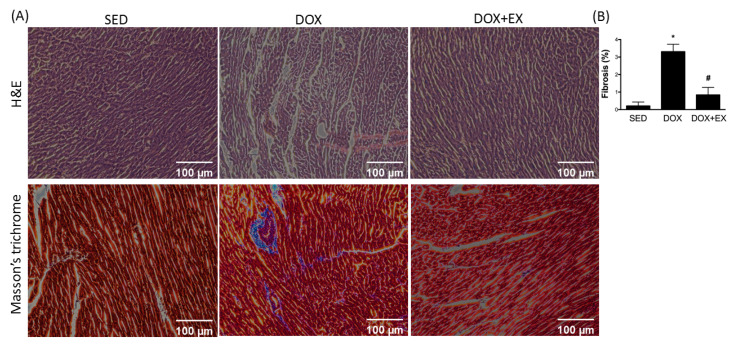
Masson’s trichrome staining of the cardiac tissues. Representative image (**A**) and quantification (**B**) of the fibrosis areas. * *p* < 0.05 compared to the SED group. # *p* < 0.05 compared to the DOX group.

Aside from the alteration at the molecular level, we also assessed the morphological change in the heart receiving DOX. As shown in Figure 4, there was an increase in collagen accumulation in the DOX-treated heart by using Masson’s Trichrome staining, and the exercise intervention inhibited the excessive collagen deposition.

**Figure 5:**
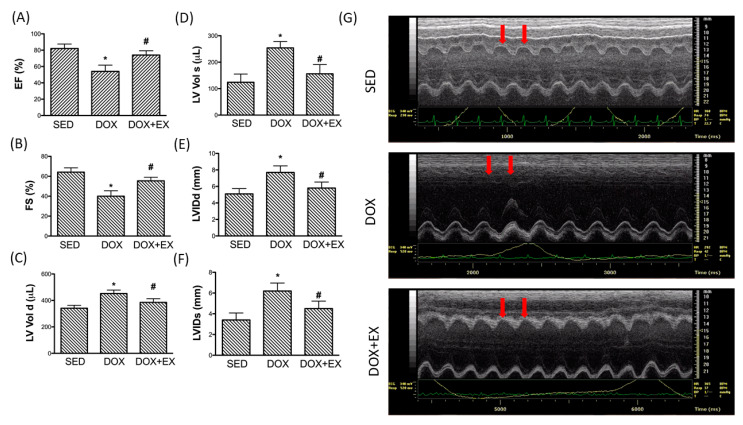
Examination of cardiac function using echocardiography: (**A**) ejection fraction; (**B**) fractional shortening; (**C**) left ventricular end-diastolic volume; (**D**) left ventricular end-systolic volume; (**E**) left ventricular internal dimension in end-diastole; and (**F**) left ventricular internal dimension in end-systole; (**G**) Representative echocardiographic M-mode images from animals. * *p* < 0.05 compared to the SED group. # *p* < 0.05 compared to the DOX group.

As for cardiac function, the echocardiographic assessment revealed that the values of ejection fraction (EF) and fraction shortening (FS) were significantly reduced in the DOX-injected heart (Figure 5A,B), but treadmill exercise preserved EF and FS. Besides, the increased left ventricular end-diastolic and end-systolic volume (LV vol d and LV vol s), as well as the left ventricular internal dimensions (LVIDd and LVIDs), were not present in the exercise+ DOX group (Figure 5C–F). Representative echocardiographic M-mode images from animals are presented in Figure 5G. 

**Figure 6:**
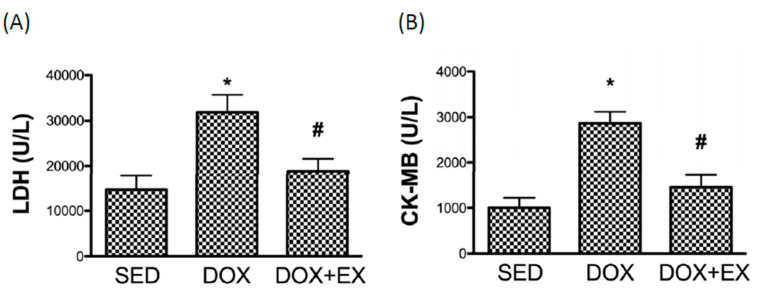
The effect of treadmill exercise on the concentration of two biomarkers of cardiac damage. The concentration of (**A**) lactate dehydrogenase (LDH) and (**B**) creatine kinase-MB (CK-MB) in SED, DOX, and DOX+EX groups in blood. * *p* < 0.05 compared to the SED group. # *p* < 0.05 compared to DOX group.

In agreement with these findings, we found that the concentration of lactate dehydrogenase (LDH) and creatine kinase-MB (CK-MB) in blood, enzymes found primarily in heart muscle cells, were both elevated after DOX treatment. These two factors are commonly used to indicate and predict myocardial injury and infarct size [26,27], and we showed that treadmill exercise was an effective intervention to reduce the damage caused by DOX (Figure 6A,B).

## 3. Discussion 

Over the past decades, a large body of research has focused on the molecular mechanisms and therapeutic approaches of DOX-associated cardiomyopathy. It has been well-accepted that DOX-induced oxidative stress resulted in alterations of mitochondrial dynamics and the cumulative cardiotoxicity caused by DOX predisposed to initiate inflammation and cardiac remodeling, which may eventually lead to tissue fibrosis and heart failure. In light of the cardioprotective effect of exercise, we conducted the present study to investigate the benefits of exercise in the modulation of cardiac damage. Exercise before and during the course of DOX treatment has been shown to limit cardiac mitochondrial-driven apoptotic signaling, regulate auto/mitophagy, and prohibit oxidative damage in DOX-treated rodents [8,28]. In breast cancer subjects, exercise performed prior to every treatment prevented changes in hemodynamics, including cardiac output, resting heart rate, and systemic vascular resistance, end-diastolic and stroke volumes [29,30] as well as attenuation of the increased NT-proBNP, a diagnostic biomarker for cardiac dysfunction [30]. In alignment with these results, we found that exercise intervention during the DOX treatment prevented cardiac functional impairment, including the reduced EF and FS, and the increased left ventricular end-diastolic and end-systolic volumes. Besides, we showed that exercise suppressed the elevation of other indicators of cardiac injury, LDH and CK-MB. An increase in LDH and CK-MB levels often represents their leakage from the damaged membranes of cardiomyocytes into the circulation [26,27], and our results demonstrated that exercise might be favorable in terms of preserving cardiac function for patients taking DOX.

AKT activation has been recognized to enhance cardiac survival via apoptosis inhibition and oxidative stress mitigation. DOX-treated cardiomyocytes have been attributed to the inhibition of AKT, thereby activating p38 MAPK and NFκB pathways [31]. Moreover, subsequent to the accumulation of oxidative stress, the inflammatory response in the heart has been attributed to the activation of the NF-κB pathway [7]. It has been demonstrated that voluntary wheel running normalized the aortic IKK and NF-κB activation, proinflammatory cytokine expression, and vascular function in old mice [32]. Exercise training has been shown to elevate AKT phosphorylation in the dilated heart with cardiomyopathy [33] and to attenuate the isoprenaline-stimulated NF-κB, TNF-α, IL-6, and TGF-β1 in the myocardium [34]. In chronic kidney disease, exercise lowered phospho-IκB, NAD(P)H oxidase, PAI-1, MCP-1, and COX-2 abundance in the cardiac tissues [35]. We demonstrated that exercise increased AKT phosphorylation and downregulated the increased expression of phospho-IκBα, NF-κB, COX-2, IL-8, and TNF-α in DOX-treated animals. Given that inflammation often drives the progression of fibrosis, this observation coincided with the results of lower expression of fibrosis markers in the exercise group. 

DOX provokes fibrosis in the myocardium [2]. The proinflammatory events are by p38 and have been thought of as a critical pathway in the development of cardiac fibrosis [36]. In addition, it has been revealed that the expression of TGF-β1 was increased in the DOX-treated hearts [37]. Approaches that were used to reduce TGF-β1 also resulted in lower interstitial and perivascular fibrosis, along with less left ventricular dysfunction [38]. It has been revealed that mitochondrial reactive oxygen species-induced ERK1/2 activation was required for DOX-induced apoptosis and cardiotoxicity [39]. Although there was no direct evidence suggested that the ERK pathway was involved in the DOX-stimulated fibrosis, a couple of studies have shown that treatments that improved cardiac function and reduced myocardial fibrosis were accompanied by decreased phospho-ERK [40,41], which were consistent with our findings. Furthermore, we demonstrated that the increased expression of Sp1 by DOX was ameliorated through exercise intervention. The transcription factor Sp1 has long been known to regulate TGF-β1 and TGF-β3 [42], and the synergistic cooperation between Sp1 and Smad3/4 was implicated in the TGF-β-stimulated type I collagen biosynthesis [22]. In associated with these results, we showed that the CTGF was upregulated by DOX, and exercise perturbed this elevation. CTGF has been revealed to mediate TGF-β1-induced fibroblast collagen synthesis [43] and was induced by TGF-β in cardiac fibroblasts and myocytes [44]. It has been demonstrated that the expression of CTGF was increased in the DOX-treated hearts, and was associated with the regulation of cardiac fibrosis and heart failure [23]. Hence, we observed an upregulation of TGF-β1 coincident with an increase in CTGF following DOX treatment, and the increased expression of these fibrosis-associated factors may contribute to the cardiac fibrosis and left ventricular dysfunction that we demonstrated. 

Cardiac remodeling often precedes DOX-induced fibrosis and heart failure. Our results showed that FGF2 was upregulated after DOX stimulation, while exercise prevented this change. Endogenous FGF2 is known to be composed of 70% high- and 30% low-molecular-weight isoforms, and Hi- and Lo-FGF2 exerted opposite biological effects. Hi-FGF2 elicited hypertrophy following myocardial infarction [45], but Lo-FGF2 protected cardiomyocytes from damage [46]. It has been shown that human Hi-FGF2 exacerbated deleterious responses, including proinflammatory, pro-fibrotic, and pro-hypertrophic events, in human atrial tissue-derived primary myofibroblasts, which may interfere with proper cardiac remodeling [47]. Antibodies that were used to neutralize human Hi-FGF2 also mitigated DOX-induced injury of cardiomyocytes [48]. We showed that suppression of FGF2 via exercise diminished the cardiotoxicity as well. However, no differences in Lo-FGF2 expressions were found in SED, DOX, and DOX+EX animals in our study model. The Lo-FGF2 might be more critical in ischemia-induced cardiac injuries [49] but not in DOX-caused cardiac dysfunction. 

In addition to FGF2, uPA and MMPs were both involved in cardiac repair and remodeling [25]. Remodeling of the myocardial extracellular matrix requires proteolysis, and various studies have shown that MMP-2 and MMP-9 are prominently overexpressed after myocardial infarction [50,51]. It has been demonstrated that MMP-9 knockout mice have less collagen accumulation in the infarcted area [50]. As a non-MMP proteinase, u-PA has been shown to be associated with the hypertension-induced cardiac fibrosis [52]. Besides, suppression of uPA, MMP-2, and MMP-9 have been demonstrated to attenuate the remodeling and dysfunction of the left ventricle after acute pressure overload [53,54] or viral myocarditis [55]. Brandon et al. suggested that MMP-2 up-regulation is an early response in DOX cardiotoxicity and leads to myofilament lysis through proteolyzing cardiac titin [56]. On the other hand, a previous study reported that inhibition of MMP-2 activity leads to cardiac fibrosis in diabetic cardiomyopathy [57]. Thus, the role of MMP-2 in cardiac cardiomyopathy is still controversial. However, studies are inclined to deem MMP-2 as a pro- cardiomyopathic marker in the DOX-treated model. DOX causes cardiac MMP-2 activation through the up-regulation of MAP kinase, and oxidative stress had been confirmed [58]. Our findings demonstrated that exercise intervention prevented the increased expression of uPA, MMP-2, and MMP-9, leading to the ameliorated myocardial remodeling and a better cardiac function. We also found treadmill training reduced p-38 activation in DOX-treated animals.

Some limitations should be discussed in this present study. First, we did not study the effects of exercise alone in this model. The over-dosage of exercise training might induce cardiac hypertrophy. However, the protocol of exercise training used has been published and has cardiac-protective effects without significant side-effects [59]. Thus, we believe this protocol is safe in clinical implications. In addition, exercise causes cardiomyocyte proliferation, thereby inducing cardiac hypertrophy and modulating cardiac fibrosis [60]. We did not confirm the effects of exercise alone on these cardiac responses in this study. These issues will be a major part of our further study. 

This present study demonstrates that real-time treadmill exercise training has positive effects on DOX-impaired cardiac function. This conclusion is supported by previous studies; for example, Duarte et al. reported that moderate endurance exercise intervention mitigates DOX-induced mitochondriopathy as well as inhibiting the up-regulation of pro-apoptotic events [61]. Precondition aerobic exercise training prevents DOX-caused impairment of cardiac function and restores DOX-reduced exercise capacity [62]. Our conclusion provides new evidence that exercise still has positive effects on cancer treatment if they have not been preconditioned to exercise training. 

Taken together, this present study revealed that treadmill exercise during the DOX treatment successfully prevented cardiac dysfunction and mitigated the expression of several cardiac remodeling-associated factors. The conclusion from this study suggested that it may be beneficial to prescribe treadmill exercise as an adjunct therapy to limit cardiac damage caused by DOX.

## 4. Materials and Methods

### 4.1. Animal Model 

All of the animal investigations followed the guidelines that were required for the care and use of laboratory animals and were approved by the animal center of the National Cheng Kung University in Tainan, Taiwan (Approval No. 107276). A total of 24 Sprague Dawley (SD) male rats of 220 ± 20 g weight were used in this study (8 in each group). After one week of acclimation in the animal center, the rats were randomly distributed into 3 groups (*n* = 8 per group): sedentary control animals (SED), DOX injection animals (DOX), and DOX + exercise intervention animals (DOX+EX).

### 4.2. DOX Injection

DOX was bought from Sigma–Aldrich (D1515, Sigma–Aldrich, USA). Phosphate-buffered saline (PBS) was used as a solvent. The final concentration of DOX stock was 1 mg/mL. The DOX stock was prepared freshly for each injection; 20 mg/kg DOX was injected to the DOX group and EOX+EX group via intraperitoneal injection 3 times per week. The equal volume of the solvent was injected in SED animals. There was a total of 5 weeks of DOX injection in this present study. 

### 4.3. Exercise Protocol

**Figure 7:**
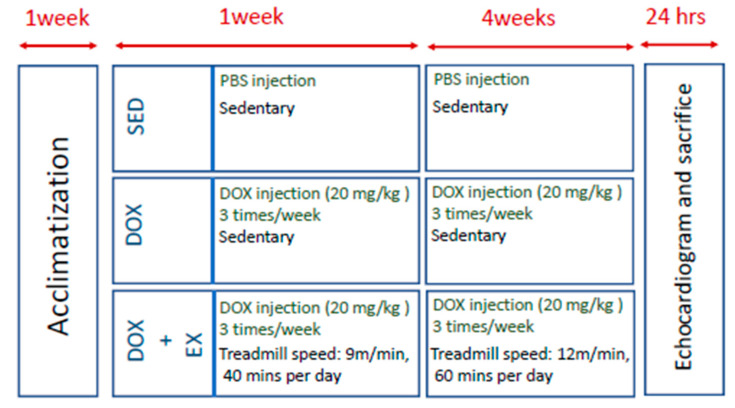
Schematic of the experimental paradigm After one week of acclimatization, all animals were assigned to three groups: sedentary group (SED), DOX-receiving group (DOX), and exercise during DOX treatment group (DOX+EX). All animals were subjected to echocardiography 24-h after the last intervention.

The exercise protocol was an involuntary moderate-intensity aerobic exercise. In the first week of acclimation, rats were placed on a nonmoving treadmill. In the next week, 20 mg/kg DOX (3 times/week) was injected into the animals. The animals were exposed to the same exercise regime (40 min/day at 9 m/min for 7 days) to get them familiarized with the treadmill. In the next step, animals were trained with 60 min at 12 m/min of treadmill exercise per day for 28 days. In this period, they still received DOX injection (20 mg/kg DOX, 3 times/week) (Figure 7). This protocol suggested the protection of cardiac dysfunction in vivo ischemia/reperfusion injuries without adverse events [59]. 

### 4.4. Tissue Extraction and Western Blotting Assay 

After sacrifice, the hearts of all the animals were collected. The tissue of right ventricles was washed 2 times with PBS buffer, and then 100 mg of tissue was cut for homogenization with RIPA lysis buffer. The homogenates were centrifuged at 13,000× *g* for 30 min, and the supernatant was collected and placed at −80 °C. until use. The proteins were transferred to a polyvinylidene difluoride (PVDF) membrane after the proteins were separated by electrophoresis on an SDS-polyacrylamide gel. The membranes were blocked by buffer for 1 h at 37 °C. Then, the membranes were incubated with primary antibodies 18 h at 4 °C followed by hybridization with HRP-conjugated secondary antibodies for 1 h. The intensities were quantified by densitometric analysis. Plasma was obtained through blood collection for LDH and CK-MB assay.

### 4.5. Determination of Cardiac Functional Parameters

Isoflurane-anesthetized animals were placed in a supine position. Echocardiographic data were collected by a Vevo 770 microimaging system with a 25-MHz probe (VisualSonics, Toronto, ON, Canada). Parameters were collected based on the M-mode and two-dimensional images obtained in the parasternal long and short axes at the level of the papillary muscles.

### 4.6. Histological Determination

The ventricular tissues were placed into 4% formaldehyde for perfusion fixation. The ventricular slice of myocardium was embedded in paraffin. Samples were embedded in paraffin, sectioned, and stained with H&E. A Masson’s trichrome staining was used for the investigation of histologically fibrotic changes. The total ventricular area and the area of fibrotic changes were assigned numerical values, and the fibrotic changes were normalized by the left ventricle.

### 4.7. Antibodies 

Anti-p-I-κBα, anti-p-p38, anti-p-NF-κB, anti-β-actin, anti-COX-2, anti-IL-8, anti-TGFβ1, anti-p-ERK, anti-SP1, anti-CTGF, anti-FGF2, anti-uPA, anti-MMP-2, and anti-MMP-9 were obtained from Cell Signaling (Danvers, MA, USA). All secondary antibodies obtained from BD Biosciences (Franklin Lakes, NJ, USA).

### 4.8. ELISA Assay 

ELISAs were performed using commercial kits according to the manufacturer’s instructions. The phosphorylated AKT and TNF-α kits were obtained from Thermo. The collagen type I and α-SMA kits were purchased from Novus. In brief, the antibody in coating buffer was added to individual wells and incubated for 2 h at 37 °C. After incubation, the coating solution was removed, and wells were washed with PBS-0.05% Tween20 twice. 100 μL blocking buffer was loaded in each well for 1 h at 37 °C. After blocking, wells were washed with PBS-0.05% Tween20 twice. 50 µL of diluted antibody was added to each well for incubation 1 h. Next, 50 µL of conjugated secondary antibody was added to each well for incubation 1 h. The absorbance of the reader was 450. 

### 4.9. Statistical Analysis 

The data are expressed as mean ± SD. One-way ANOVA, followed by Student’s *t*-test, was used to analyze the differences between groups. In all cases, * *p* < 0.05 be considered as significant.

## 5. Conclusions

Overall, our results suggested that treadmill exercise may serve as an effective nonpharmacologic strategy to decrease the NF-κB and ERK activation along with the downregulation of inflammatory molecules (COX-2 and IL-8) in the DOX-treated hearts. Also, exercise intervention lowered the expression of various fibrosis factors, such as TGF-β1, Sp1, and CTGF, which supported our observation of less cardiac fibrosis and the reduced expression of remodeling-associated factors (FGF2, uPA, MMP-2, and MMP-9). The downregulated inflammation and fibrosis by treadmill exercise improved the DOX-induced morphologic and echocardiographic changes. These findings suggested that exercise prescription may be an advantageous intervention to limit DOX-induced cumulative cardiotoxicity.

## Supplementary Materials

The following are available online at https://www.mdpi.com/2072-6694/12/5/1102/s1, Figure S1: Detailed information about Figure 2B, Figure S2: Detailed Information about Figure 3A, Figure S3: Detailed information about Figure 4A.

## Abbreviations

doxorubicin (DOX); nuclear factor kappa B (NF-κB; extracellular signal-regulated kinase (ERK1/2); I-κB kinase (IKK); cyclooxygenase-2 (COX-2); fibroblast growth factor 2 (FGF-2); connective tissue growth factor (CTGF); urokinase-type plasminogen activator (uPA); matrix metalloproteinases (MMPs)
