# Supplementary material for: Early Moderate Intensity Aerobic Exercise Intervention Prevents Doxorubicin-caused Cardiac Dysfunction through Inhibition of Cardiac Fibrosis and Inflammation
Source: Cancers (Basel). 2020 Apr 28;12(5):1102. doi: 10.3390/cancers12051102 (PMC7281105; doi:10.3390/cancers12051102)
Supplement: Supplementary file 1 [file cancers-12-01102-s001.pdf]

Supplementary Figures

Early Moderate Intensity Aerobic Exercise Intervention Prevents Doxorubicin-caused Cardiac Dysfunction through Inhibition of Cardiac Fibrosis and Inflammation

Hsin-Lun Yang, Pei-Ling Hsieh, Ching-Hsia Hung, Hui-Ching Cheng, Wan-Ching Chou, Pei-Ming Chu, Yun-Ching Chang and Kun-Ling Tsai

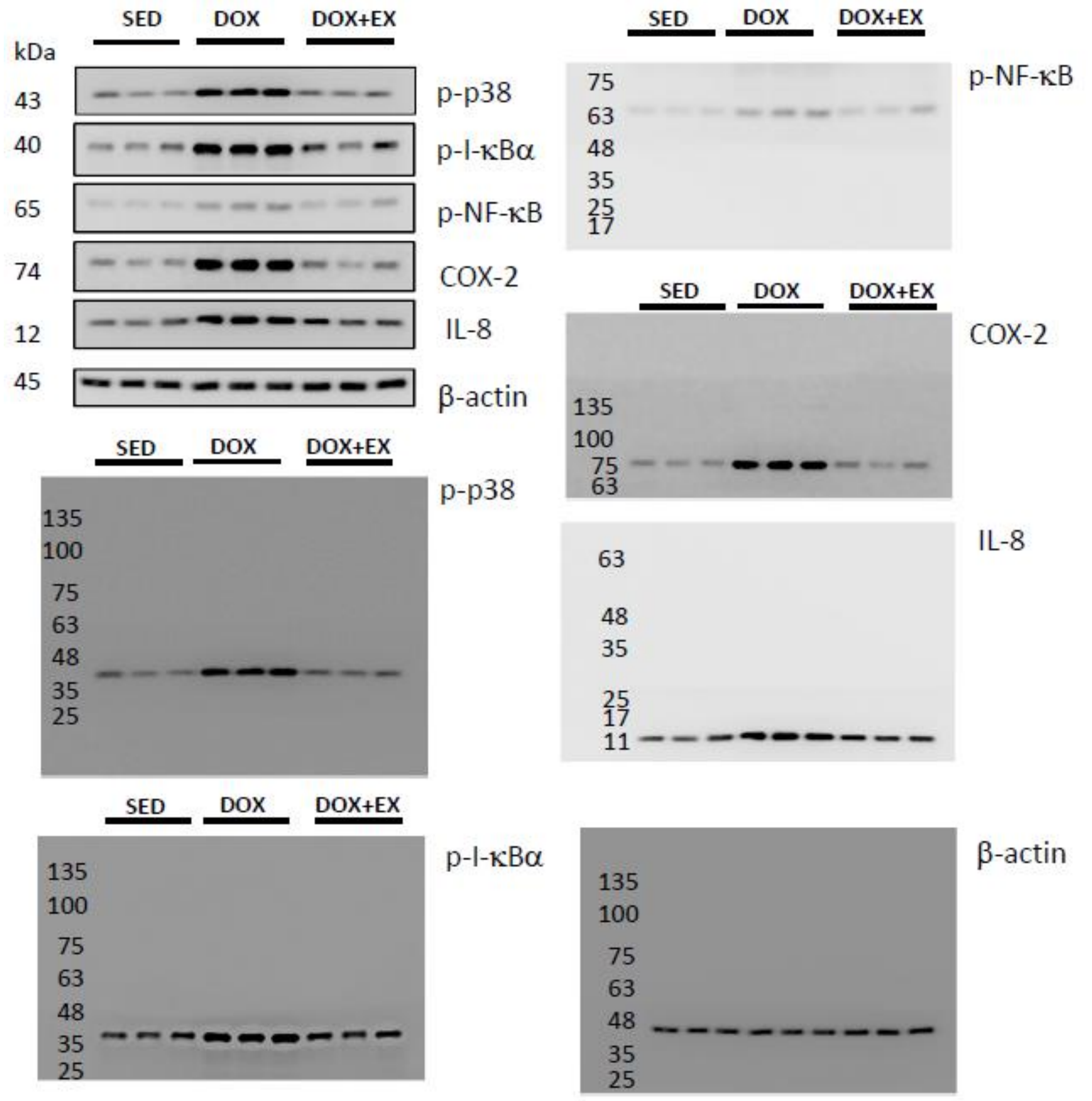

Figure S1. Detailed information about Figure 2B.

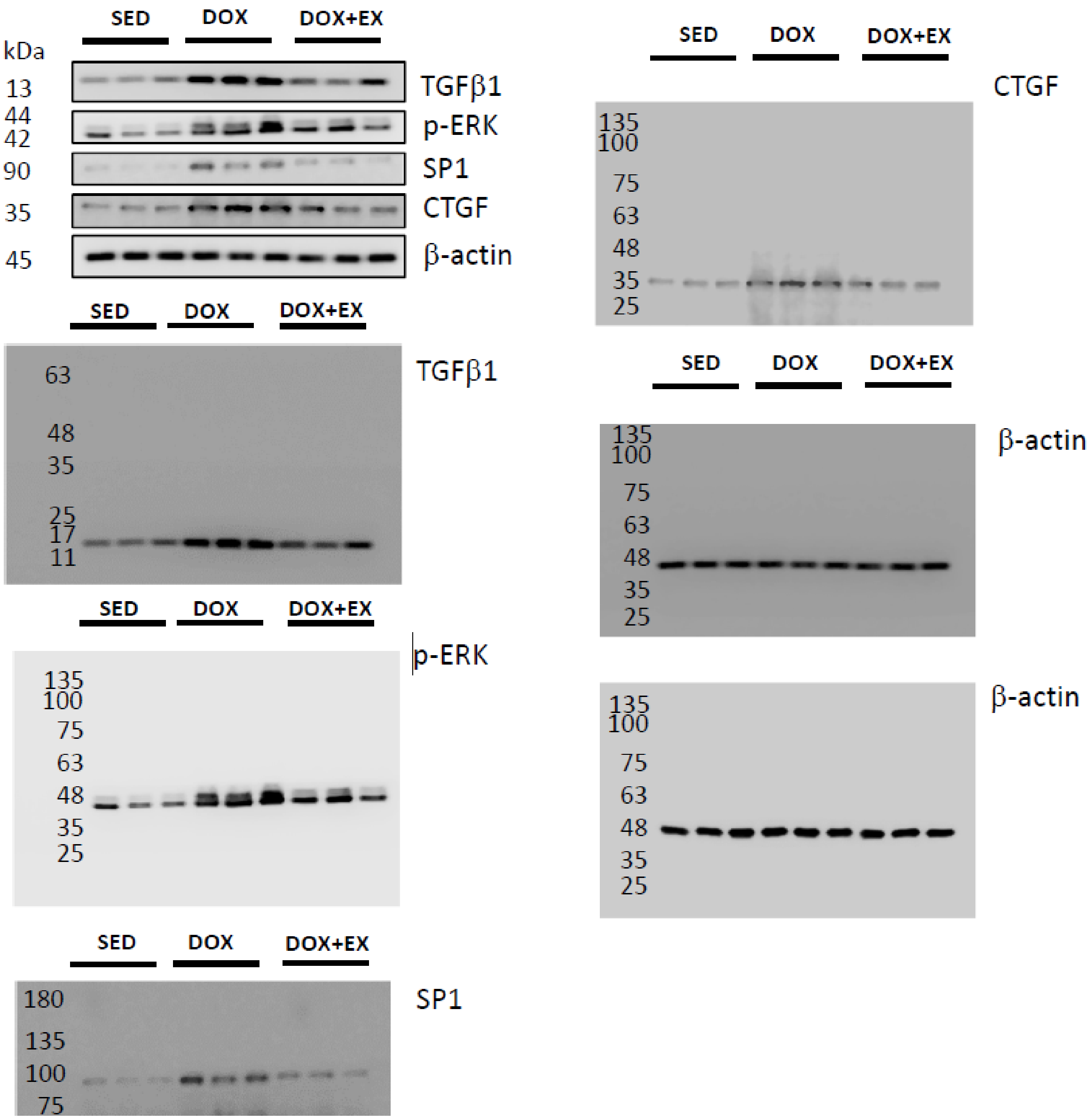

Figure 2. Detailed Information about Figure 3A.

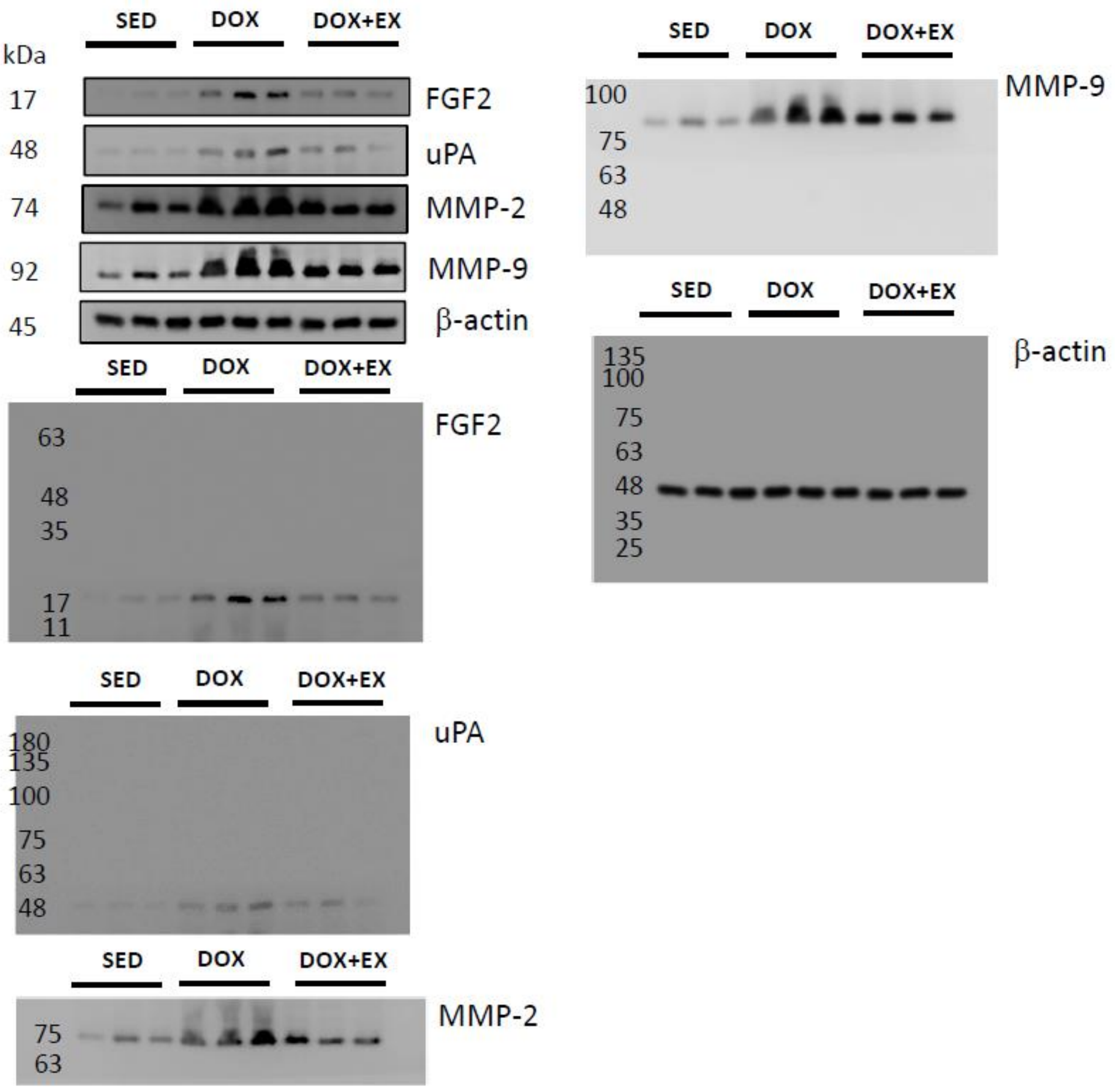

Figure 3. Detailed information about Figure 4A.

© 2020 by the authors. Licensee MDPI, Basel, Switzerland. This article is an open access article distributed under the terms and conditions of the Creative Commons Attribution (CC BY) license (http://creativecommons.org/licenses/by/4.0/).
